# Marine Bioactives and Potential Application in Sports

**DOI:** 10.3390/md12052357

**Published:** 2014-04-30

**Authors:** Maria Alessandra Gammone, Eugenio Gemello, Graziano Riccioni, Nicolantonio D’Orazio

**Affiliations:** 1 Human and Clinical Nutrition Unit, Department of Biomedical Science, Via Dei Vestini, University G. D’Annunzio, Chieti 66013, Italy; E-Mails: egemello@libero.it (E.G.); griccioni@hotmail.com (G.R.); ndorazio@unich.it (N.D.); 2 Cardiology Unit, San Camillo De Lellis Hospital, Manfredonia, FG 71043, Italy

**Keywords:** marine bioactives, oxidative stress, reactive oxygen species, antioxidants, sports

## Abstract

An enriched diet with antioxidants, such as vitamin E, vitamin C, β-carotene and phenolic compounds, has always been suggested to improve oxidative stress, preventing related diseases. In this respect, marine natural product (MNP), such as COX inhibitors, marine steroids, molecules interfering with factors involved in the modulation of gene expression (such as NF-κB), macrolides, many antioxidant agents, thermogenic substances and even substances that could help the immune system and that result in the protection of cartilage, have been recently gaining attention. The marine world represents a reserve of bioactive ingredients, with considerable potential as functional food. Substances, such as chitin, chitosan, *n*-3 oils, carotenoids, vitamins, minerals and bioactive peptides, can provide several health benefits, such as the reduction of cardiovascular diseases, anti-inflammatory and anticarcinogenic activities. In addition, new marine bioactive substances with potential anti-inflammatory, antioxidant and thermogenic capacity may provide health benefits and performance improvement, especially in those who practice physical activity, because of their increased free radical and Reacting Oxygen Species (ROS) production during exercise, and, particularly, in athletes. The aim of this review is to examine the potential pharmacological properties and application of many marine bioactive substances in sports.

## 1. Introduction

**Figure 1:**
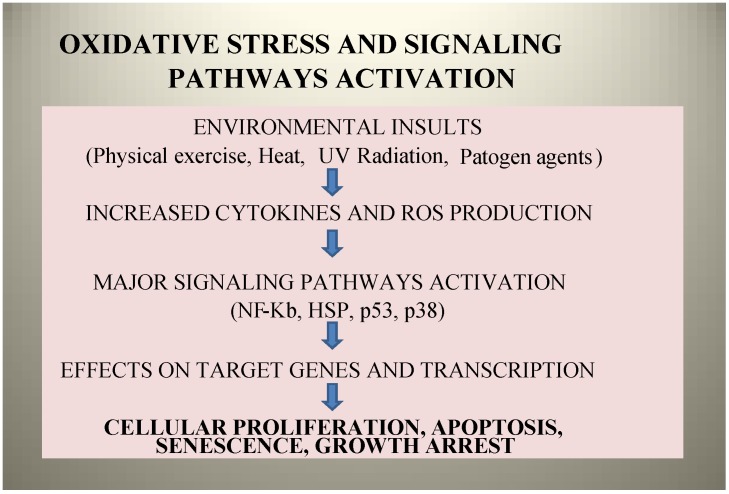
Reacting Oxygen Species (ROS) enhance oxidative reactions with proteins, lipids and DNA: oxidative stress activates signaling pathways and can impair cellular functions, causing secondary damage. ROS accumulation can decrease an organism’s fitness, because oxidative damage is a contributor to fatigue and senescence.

Free radicals are unstable molecules oxidizing other molecules in order to become stable. In basal conditions, the skeletal muscle produces Reacting Oxygen Species (ROS) at a low rate, represented in particular by superoxide anions, mainly at the mitochondrial Complex III. However, during contractile activity, this production increases. In fact, aerobic exercise increases oxygen consumption, especially by the contracting muscle, with an increase of 15-fold in the rate of whole body oxygen uptake and an increase of more than 100-fold in the oxygen flux in active muscles [1]. ROS production by contracting muscle during exercise happens through several mechanisms: the activation of endothelial xanthine oxidase, electron leak at the mitochondrial electron transport chain, inflammatory response and increased release and autoxidation of catecholamines, determining a depletion of cellular antioxidant concentration in the blood, such as glutathione, altering the oxidation-reduction balance [2]. As a consequence, exercise leads to the upregulation of the body’s antioxidant defense mechanisms, in order to minimize the oxidative stress. Free radicals can have useful roles in cells: ROS are produced by immune cells in order to eliminate antigens [3] and stimulate signals of several genes encoding transcription factors involved in cell proliferation and antioxidant enzyme expression. However ROS and other oxidants enhance oxidative reactions with proteins, lipids and DNA [4], and this oxidative stress can impair cellular functions, causing secondary damage, such as lipid peroxidation (Figure 1). Oxidants have been directly linked to the stimulation of inflammation genes in endothelial cells. Similarly, ROS have been attributed to an aggravating role in inflammation that accompanies asthma and exercise-induced muscle damage [5]. In fact, during exercise, ROS production can be higher than antioxidant capacity. As ROS accumulates in contracting muscles, protein and lipid oxidation might inhibit force production, contributing to the development of acute fatigue [6]. In addition, oxidative DNA modification might inhibit the locomotory and bactericidal activity of both neutrophils and natural killer cells and T- and B-lymphocytes proliferation [7]. This negative action of reactive species and free radicals in lipid, protein and DNA damage has led researchers to analyze the efficacy of dietary antioxidant supplementation. Vitamin and mineral supplements are often used by athletes as ergogenic aids to improve performance: in particular, vitamin E has been found to protect cellular membranes from lipid peroxidation, protecting muscle cells against exercise-induced damage. In the same way, antioxidants deriving from marine natural products (MNPs) could also bring benefits to athletes in order to attenuate muscle oxidative stress generation and, thus, improve muscular performance and immune function [8].

## 2. Marine Carotenoids as Antioxidant and Thermogenic Agents

The great potential of marine compounds, as such or as extracts, for applications in different areas, such as human nutrition, as anti-inflammatory, antiallergic and analgesic agents, is gaining more attention in the literature. Habitual intake of marine fish and seafood, such as microalgae, which are very rich in some chemical compounds, has been strongly associated with several benefits in human health (Table 1). It was shown to promote cardiovascular health and to enhance immune competence, preventing infectious diseases [9]. Another possible reason for marine bioactives’ potential success may be their thermogenic effect. This thermogenic effect of some foods consists in the increase both in energy expenditure above the baseline, following their consumption, and the energy required for the digestion, absorption and disposal of the ingested nutrients. The effect on thermogenesis seems to be influenced by consumed food composition: for example, the typical thermic effect of protein is 20%–35% of the energy consumed, and for carbohydrate it is 5%–15% [10], so diets higher in protein were shown to exert a larger effect on energy expenditure than diets lower in protein. The thermogenic capacity of some food could become clinically significant with chronic consumption, because it increases daily energy expenditure, contrasting weight gain. In addition, marine bioactives have also a great antioxidant capacity. Some studies [11] reported that diets high in carotenoids are associated with a reduced risk of developing inflammatory polyarthritis (IP). In particular, beta-cryptoxanthin and probably zeaxanthin intake may have a stronger association with the onset of joint inflammation than other dietary carotenoids. The strongest relation was observed between beta-cryptoxanthin intake and inflammatory polyarthritis (IP) onset, which supports the finding from the Iowa Women’s Health Study of an inverse association between high carotenoids level and the risk of IP development [11]. The outcome of interest in this study was the decreased risk of developing IP and rheumatoid arthritis (RA) thanks to some terrestrial carotenoid, but our hypothesis is that some antioxidant nutrients present also in marine carotenoids may protect against inflammatory arthritis and musculoskeletal inflammation and, thus, all cases that are sports related could be included.

Antioxidants, carotenoids in particular, protect cells against oxidative damage, but they are implicated in regulating gene expression and in cell-to-cell signaling [12]. Carotenoids are present in plants, algae and microorganisms; they are able to bind heavy metals and toxic substances, preventing their accumulation in humans [13]. Unfortunately, humans are not able to synthesize carotenoids and require them as part of their diets. There are two classes of carotenoids: carotenes and xanthophylls. The major marine carotenoids are astaxanthin and fucoxanthin, whose strong antioxidant activity is attributed to quenching single oxygen atoms and scavenging free radicals [14].

**Table 1:** The health applications of some marine bioactive compounds.

Group	Bioactives	Source	Activity	Health Application
**Pigments**	β-Carotene	*Dunaliella salina*	Pro-vitamin A; antioxidant	Food supplement [15,16]
	Astaxanthin	*Haematococcus* *pluvialis*	Anti-inflammatory; antioxidant	CTS and muscle soreness [5,15,17,18,19,20,21,22,23]
	Fucoxanthin	*Undaria pinnatifida*	β-oxidation and UCP upregulation	Increase in energy release from fat [24,25,26,27,28]
	Lutein, zeaxanthin	*Chlorella* *pyrenoidosa*	Antioxidant	Food supplement [15,29]
	Phycocyanin	*Spirulina*	Anti-inflammatory; antioxidant	Muscle soreness [15,29]
**Sterols**	Stigmasterol	*Chaetoceros*	Hypocholesterolemic	Dyslipidemia [15,30]
	Contignasterol	*Petrosia contignata*	Anti-inflammatory; antiallergic	Asthma/inflammatory diseases [31,32,33,34,35,36,37]
	Xestobergsterol	*Xestospongia bergquistia*	Anti-inflammatory; antiallergic	Asthma/inflammatory diseases [38,39]
	Clathriols	*Clathria lissosclera*	Anti-inflammatory	Inflammatory diseases [40,41,42,43]
**Vitamins**	C, K, B12, A, E	*Arthrospira*	Antioxidant; blood cell formation	Immune system reinforcement [15,44]
		*Pavlova*	and blood clotting mechanisms	
**Proteins**		*Dunaliella*	Anti-catabolic	Muscular status and
		*Arthrospira platensis*		performance improvement [15]
**Enzymes**	SOD	*Anabaena*	Antioxidant; anti-inflammatory	Food supplement [15,44]
	Carbonic Anhydrase	*I. galbana*	CO_2_ conversion into H_2_CO_3_/HCO_3_	Muscular performance improvement [15]
**PUFA**	EPA	*Odontella, Pavlova*	Antimicrobial;	Immune system improvement [45,46,47,48,49,50,51,52,53,54,55]
	γ-Linolenic acid	*Arthrospira*	Strongly anti-inflammatory	Tissues integrity; delay of aging
**Others**	Pacifenols	*Laurencia claviformis and tasmanica*	COX-inhibitor	Anti-inflammatory [56,57,58,59,60]
	Epitaondiol	*Stypopodium flabelliforme*	COX-inhibitor; negative inotropism	Anti-inflammatory; reduced cardiac stress [61,62,63,64,65,66]
	Cycloprodigiosine	*Serratia marcescens*	NF-κB inhibitor; NO stimulator	Anti-inflammatory/anti-arthritic [67,68,69,70,71]
	Macrolides	*Stylocheilus longicauda*	NF-κB inhibitor; antibiotical effect	Immunomodulation; Anti-inflammatory [72,73]
	GABA	*Porphyridium*	Neurotransmitter; antioxidant [15,74]	CNS regulation; immune improvement
	Hymenialdisine	*Stylissa massa*	Inhibition of proteoglycan degradation	Decrease in joint injuries risk [75,76,77,78]
	Stypotriol	*Stypopodium* f*labelliforme*	PA2 and elastase release inhibition	Anti-inflammatory; less cartilage damage [63,79]

Abbreviations: CTS, carpal tunnel syndrome; SOD, superoxide dismutase; EPA, eicosapentaenoic acid; COX, cyclooxygenase; CNS, central nervous system; PA2, phospholipase A2; PUFA, polyunsaturated fatty acid.

### 2.1. Astaxanthin

Astaxanthin is the main carotenoid pigment found in the chlorophyte alga, *Haematococcus pluvialis*, and in aquatic animals, especially in some popular seafood, such as shrimp, trout, salmon, lobster and fish roe [80]. Astaxanthin possesses powerful antioxidant and free radical scavenging activities, even stronger than vitamin E and β-carotene, because it contains two additional oxygenated groups on each ring than other carotenoids [17] and several essential biological functions. It protects against UV light effects, contrasts inflammation, aging and age-related diseases and promotes the immune response in the heart, eyes liver, kidney and joints. Astaxanthin protects membranous phospholipids from peroxidation (which is well known to be higher after physical exercise), and it resulted in being associated with shifts in inflammatory response [18]. The effects of the combination of astaxanthin and fish oil (FO) are summative and synergistic: FO components, well known as *n*-3 polyunsaturated fatty acids (PUFA), are antioxidant compounds, which significantly diminish superoxide (O-2) and hydrogen peroxide (H_2_O_2_) production in activated neutrophils [19]. In addition, they increase anti-inflammatory responses by stimulating anti-inflammatory interleukin production and phagocytic activity in activated neutrophils [20]. Astaxanthin offers antioxidant/anti-apoptotic effects, through glutathione redox balance improvement. Thus, habitual consumption of marine fish, such as salmon, which is a natural source of both astaxanthin and fish oil, is associated with immune response improvement and lower risks for vascular and infective diseases [21]. This results in being helpful, especially to athletes, whose intense exercise causes not only a greater ROS production, but also an immune reaction to stress: in particular, in the case of excessive training, there is a greater susceptibility to infections, for temporary immunosuppression. Clinical studies also demonstrated reductions in the cardiovascular risk markers of oxidative stress and inflammation, improved blood status [22] and a potential in the prevention of various chronic inflammatory disorders, such as cancer, arthritis, metabolic syndrome, diabetes and also gastrointestinal, liver and neurodegenerative diseases [23]. An improvement as a result of *Haematococcus* astaxanthin supplementation was observed in 88% of the considered health conditions, such as sore muscles and joints or back pain. In comparisons with popular brands of prescription drugs, *Haematococcus* astaxanthin supplementation was reported to be as effective as or more effective than the anti-inflammatory drugs in 92% of the comparisons. Of 62 comparisons with aspirin or ibuprofen, astaxanthin supplementation was reported as effective or more effective in 76% of the comparisons [5]. This nutritional supplementation has also been valued as a potential adjunct in the conservative management of carpal tunnel syndrome (CTS) [15]. Therefore, its daily consumption is a beneficial strategy in human health management, and in particular, it could result in being successful in fighting oxidative stress in athletes, whose free radical production is accentuated, because of physical exercise.

### 2.2. Fucoxanthin

Fucoxanthin is a brown pigment belonging to the class of xanthophylls, with antioxidant properties (even under anoxic conditions) and free-radical quenching functions through electrons cession [24]. During normal metabolism, the human body produces heat: fucoxanthin affects many enzymes involved in fat metabolism, determining an increase of thermogenesis and an increased release of energy from fat [25]. Some human overfeeding studies [26] support the view that in diets with increasing thermogenesis, there is an effort to homeostatically waste energy: an increase in thermogenesis ensures an adequate supply of nutrients, avoiding the risks associated with excess weight gain. The increased amount of energy attributable to this thermic effect may significantly increase the total weight lost, especially the percentage of fat lost in favor of lean mass, which is positively related to physical performance. In this sense, several studies showed a potential anti-obesity effect of fucoxanthin, which may be mediated by altering the plasma adipokine level, downregulating fat production, upregulating β-oxidation and UCP gene expressions in visceral adipose tissues: fucoxanthin upregulates the gene expression of the uncoupling proteins, UCP1 and UCP3, in brown adipose tissue (BAT) and UCP2 in white adipose tissue (WAT), which is a primary site of energy storage, accumulating triglycerides during nutritional excess [27]. The UCP1 in BAT explain a significant component of whole body energy expenditure: its dysfunction contributes to the development of obesity [28], and its expression would also be an attractive target for the development of anti-obesity therapies. Both athletes and coaches believe that thinness can exploit a significant effect on physical performance [81]. Studies on distance runners reported that leaner runners display better sports performance: one study reported that medal-winning gymnasts tended to have lower body fat than the non-medal-winning gymnasts [82]. Another study found a correlation between low Body Mass Index (BMI) and better performance among gymnasts participating in the world championships, but pointed out that this trend was reversed when BMI became very low [83]. This suggests that, although weight loss and low weight may enhance athletic performance in certain sports, there is a point beyond which excessive weight loss produces a negative effect on performance, presumably due to the excessive loss of fluid and lean mass. UCP2 and UCP3 are expressed in various tissues, such as BAT, skeletal muscle, WAT, lung, liver, kidney and the immune system. In particular, UCP2 and UCP3 in skeletal muscles regulate the thermogenesis in obese mice and attenuate the mitochondrial production of free radicals in cells, protecting against oxidative damage [84]. For these reasons, UCP2 and UCP3 can be important targets for the treatment of aging, degenerative diseases, diabetes and, perhaps, obesity [85]. Thus, fucoxanthin seems to enhance the thermogenic capacity of BAT and the UCP1 gene expression in WAT, acting as a regulator of lipid metabolism in fat tissues and of energy expenditure. Fucoxanthin resulted in being also a powerful antioxidant protecting cells from oxidative damage and providing other health benefits, such as improved liver function and cardiovascular health and the reduction of inflammation, cholesterol, triglycerides (TG) levels and blood pressure levels [86], preventing oxidative stress and related diseases both in athletes and in non-athletes. Future clinical studies will determine the effectiveness of these marine carotenoids (astaxanthin and fucoxanthin) on cartilage, both in osteoarthritis patients and subjects overtraining their joints and exposing themselves to cartilage wear risk, in particular athletes practicing high articular impact sports.

## 3. Marine Natural Products: A Potential Immunomodulating and Anti-Inflammatory Strategy?

Pain is a natural mechanism of protection against injuries and overuse, representing an important diagnostic feature [86]. Athletes are frequently exposed to unpleasant sensory experiences during their daily physical efforts, and high physical and psychological resistances must be overcome during competitions or very exhausting activities. Even if the mental attitude of athletes towards pain significantly differs from that of normally active controls [87] with a consistently higher pain tolerance, a large number of athletes sustain musculoskeletal injuries and inflammation. The most frequent sports injuries happens in football and handball, often involving the lower extremity, primarily consisting of distortions and ligament tears, sometimes of fractures. Ski injuries usually lead to knee problems. Spine injuries are observed most often during horse riding and head injuries in bicycle accidents [88]. The modern pentathlon can cause idiopathic injuries, because it requires a high degree of physical fitness. The technique required by each sport is very complex and differs in each sport, and this is necessary to perform static and dynamic games. Sports injuries show diversity based on the injury type and environment. They are generally accompanied with functional degradation on the peripheral of the injury, and when the consideration of this functional degradation is insufficient during rehabilitation, redamage or a decrease in athletic performance can be caused. Generally, repeated injury or incomplete recovery can cause instability and degradation of muscular strength [89]. This can affect the sports ability of the entire body and cause kinetic changes. Most anti-inflammatory drugs used against inflammation and pain are cyclooxygenase (COX) inhibitors. Analgesic effects of non-steroidal anti-inflammatory agents, such as aspirin and indomethacin, consist of inhibiting the production of prostaglandins and decreasing the sensitivity of peripheral nociceptors [90]. The enzyme, COX-1, produces prostaglandins, which protect kidney and stomach from tissue and mucosal damage: its inhibition relieves pain, but causes renal damage and gastric irritation, the typical side effects of aspirin-like drugs [91]. The other enzyme, COX-2, contributes to the inflammation, but requires more time to form prostaglandins. However, among inflammation-related targets, we should consider not only COX, but also molecules able to interfere with factors involved in the modulation of gene expression, such as NF-κB, which could also act as potential anti-inflammatory agents [31]. In this respect, marine natural bioactives were recently shown to contain not only antioxidant agents, but also steroids and many molecular entities potentially able to target COX-1, COX-2 and the NF-κB pathway [92], resulting in the helpful blocking of pain and inflammation, sometimes avoiding drug consumption. This pharmacological potential could increase athletic performance in the future and provide alternative rehabilitation program development.

### 3.1. COX Inhibitors: Pacifenol and Epitaondiol

Pacifenol, whose structure was characterized by crystallographic analysis in 1971, is a terpenoid obtained from the marine alga, *Laurencia claviformis*, in Easter Island [56]. This halogenated sesquiterpene is the first example of a natural bioactive obtained from algae containing bromine and chlorine atoms that are covalently bound in the chemical literature [57]. The precursor of pacifenol, prepacifenol, was originally isolated from the Australian red alga, *Laurencia filiformis*, but later, pacifenol was naturally found in *Laurencia tasmanica* [58]. Subsequently, these new metabolites, pacifenol and prepacifenol, were found also in some marine invertebrates, for example in the digestive system of the mollusk, *Aplysia californica* [59]. Pacifenol inhibits inflammation by decreasing leukotriene B4 (LTB4) and thromboxane B2 (TXB2) production, and it also blocks the degranulation response [60]: This anti-inflammatory action, exercised through the inhibition of the key enzyme, phospholipase A2, and the consequent modulation of the cyclooxygenase pathway and its anti-allergy effect could contrast phlogistic processes and allergic diseases. Furthermore, epitaondiol is a terpenoid, isolated from the seaweed, *Stypopodium flabelliforme*, collected near Easter Island in the South Pacific Ocean [61]. The genus Stypopodium is a tropical group of brown algae, Phaeophyceae, with rich components of polycyclic meroditerpenoids, possessing several biological activities [61]. Epitaondiol diacetate showed pharmacological effects in the rat cardiovascular system; a negative inotropic and chronotropic effect was noticed [62], but it also revealed marked anti-inflammatory effects through the inhibition of eicosanoids (LTB4 and TXB2) release and modulation of the cyclooxygenase pathway, through the inhibition of the key enzyme, phospholipase A2, which plays an important role in the release of arachidonic acid and the formation of lipid mediators [63]. It possesses both anti-inflammatory activity even stronger than indomethacin [64] and a dose-dependent gastroprotective activity in mice gastric lesions, similar to lansoprazole [65]. This strong anti-inflammatory effect could be exploited against exercise-related inflammation. In fact, exercise triggers the simultaneous increase of various antagonistic mediators. It also elevates catabolic proinflammatory cytokines, such as interleukin 1β (IL-1β) and tumor necrosis factor alpha (TNFα). On the other hand, it also stimulates anabolic components, such as interleukin 6 (IL-6), interleukin 10 (IL-10) and heat shock proteins (Hsps), which protect against stressors. If an anabolic response is stronger, training will probably, ultimately, lead to an enhanced muscle mass and improved exercise adaptation. At the same time, an excessive IL-1β and TNFα release may be responsible for the overtraining [66]. Pacifenol’s and Epitaondiol’s double effect, both anti-inflammatory and gastroprotective, could be a fascinating treatment strategy without the well-known side effects of drugs that are conventionally prescribed for the treatment of pain and inflammation. In addition, epitaondiol exhibited antimicrobial effects [93,94] and antiproliferative properties, especially against human colorectal adenocarcinoma and neuroblastoma cell lines [95]. In addition, its derivative, 2β-3α-epitaondiol, possesses sodium channel blocking activity, resulting in cytotoxicity against human lung cancer cells [96]. A similar molecule, called isoepitaondiol, showed a radical scavenging activity even more powerful than ascorbic acid [97]. These anti-inflammatory and antioxidant effects deserve further study, since they may be helpful in targeting the inflammation and oxidative stress happening both in inflammatory disease and in sports. At present, it is known that pro- and anti-inflammatory cytokine concentrations alter as a result of physical activity in a way dependent on the discipline. Ongoing studies are considering further and more specific data, including the number of matches played, their intensity and duration; in addition, it is fundamental to plan trainings in order both to emphasize the anti-inflammatory pathways and to stimulate recovery from inflammation in athletes.

### 3.2. Marine Steroids: Contignasterol, Xestobergsterol and Clathriols

Steroids are synthetic drugs widely used for treating a wide variety of inflammatory conditions, by decreasing inflammation and reducing the activity of the immune system. Their action provides relief from phlogosis-related symptoms, such as articular pain or stiffness and dyspnea. Marine sponges have recently been recognized as a source of uncommon steroids showing potent biological anti-inflammatory activities. Contignasterol is a natural polyoxygenated steroid, isolated from the marine sponge, *Petrosia contignata*, in Papua New Guinea [32]. It presents a particular chemical structure with a new side chain and with an unusual set of functional groups [33]. Study results have shown that it inhibits the release of histamine from human basophils and lung tissue and attenuates the contractile response to histamine. Probably, contignasterol indirectly interacts with cellular signaling systems, leading to the inhibition of phospholipase C activity [34]. In this way, it protects from bronchoconstriction, with a potential value in the treatment of asthma and other inflammatory diseases [31]. These effects of contignasterol could result in helping to counter exercise-induced bronchoconstriction. This term describes the narrowing of the airways during or following exercise and is associated with exercise-induced symptoms of breathlessness, wheeze and cough. It is a common symptom in asthma and is a marker of the presence of airway inflammation. However, up to 20% of individuals with exercise-induced bronchoconstriction do not have a diagnosis of asthma, so do not assume therapies [35], and, therefore, may represent a discreet clinical entity that can benefit from non-drug remedies. Dietary antioxidants, such as vitamin C, in the epithelial lining and lining fluids of the lung have been shown to result in a beneficial the reduction of oxidative damage [36]. Both terrestrial and marine antioxidant may therefore be of benefit in reducing the symptoms of inflammatory airway conditions, such as asthma, and may also be beneficial in reducing exercise-induced bronchoconstriction, which is a well-recognized feature of asthma and is considered a marker of airway inflammation. However, the association between dietary antioxidants and asthma severity or exercise-induced bronchoconstriction is not fully understood yet. In addition, contignasterol showed an anti-thrombolytic activity, through inhibiting platelet aggregation in response to the collagen exposure of vessels and to their activating factor, PAF (representing a local mediator of thrombotic events). As a consequence, the pharmacological potential of contignasterol as a cardiovascular and antiallergic drug could be exploited to treat asthma, allergic rhinitis, psoriasis, rashes, osteoarthritis, hemodynamic disorders involving platelets, hypertension or hypotension, thrombosis and inflammation in general [37]. Xestobergsterol is pentacyclic polyhydroxylated steroid, isolated in 1992 from the Okinawan marine sponge, *Xestospongia bergquisita* [38]. It inhibits IgE-mediated histamine release from activated mast cells [39], with an inhibitory effect that resulted in being even stronger than some anti-allergy drugs, such as disodium cromoglycate [33]. In detail, xestobergsterol A blocks the generation of inositol triphosphate (IP3) and phospholipase C (PLC) activity in a dose-dependent way and inhibits early events in IgE-dependent mediator release, such as Ca^2+^-mobilization from intracellular stores [38,39]. Therefore, like contignasterol, it could be considered as a potential anti-asthma agent with a promising pharmacological potential [31]. Other novel marine steroids are clathriols. In particular, clathriols A and B, isolated from the sponge, *Clathria lissosclera*, in New Zealand seas, possess the rare natural 14-β-stereochemistry [40], which makes them quite similar to contignasterol from a biological and structural point of view, but less strong in inhibiting histamine release. Both are anti-allergy and also anti-inflammatory molecules [40,41,42,43], through blocking superoxide production in human blood neutrophils [95]. As a consequence, these marine steroids may be able to interfere with the pathogenesis of inflammatory diseases and may alleviate inflammation, which often occurs in sports after trauma or overtraining, and the concomitant oxidative stress.

### 3.3. Molecules Interfering with NF-κB and Immunomodulation: Cycloprodigiosin and Marine Macrolides

Cycloprodigiosin, which belongs to the prodigiosin family [67], is a red pigment produced by various marine bacteria, such as *Pseudoalteromonas denitrificans* and *Serratia marcenses*. Cycloprodigiosin possesses apoptotic and immunosuppressive properties, because of its interference on p65 and the nuclear factor, κB (NF-κB), pathway [68]. This inhibition of the NF-κB pathway confers both immunosuppressant and anti-tumor effects [69]. Cycloprodigiosin was also shown to stimulate nitric oxide production, improving the cell status by regulating the expression of NF-κB-dependent genes, such as inducible nitric oxide synthase (iNOS) [70]. Both suppression of NF-κB (an important transcription factor regulating inflammatory response) and increased NO production have been suggested as an anti-inflammatory strategy in inflammatory bowel disease (IBD) and in rheumatoid arthritis (AR). Similarly, the administration of cycloprodigiosine may limit inflammation, which often occurs in muscle injuries during physical exercise. Anti-inflammatory and anti-arthritic properties possessed by cycloprodigiosin, together with the increased production of NO, notoriously determining useful vasodilatation, could also be exploited in athletes. Another class of marine bioactives with a good potential, both in anticancer and in rheumatologic research therapy, is represented by macrolides, because of their cytotoxic, immunosuppressant and anti-inflammatory properties. These highly oxygenated natural products, structurally characterized by a macrocyclic lactone, includes more than 200 substances, such as the aplysiatoxins, obtained from the sea hare, *Stylocheilus longicauda*, which showed immunomodulation, antiviral and antifungal properties [71]. Other macrolides, such as lobophorins A and B, isolated from an actinomycetes, found in the Caribbean brown alga, *Lobophora variegata,* showed antibiotic, anticancer and anti-inflammatory properties, even stronger than indomethacin. In fact, physiological and biochemical studies in murine inflammatory models demonstrated that these anti-inflammatory marine natural products selectively inhibit 5-lipoxigenase [72]. Their anti-inflammatory action can result in helping to counter exercise-related inflammation, due to physical stress, and repeated microtrauma, due to many kinds of sports. In non-damaging, endurance-type activities (that induce no structural and functional damage to the muscle), the stress response is thought to be mediated by redox signaling, because of transient and reversible oxidation of muscle proteins as opposed to increases in the contracting muscle temperature. On the other side, in damaging forms of exercise, the stress response is initiated by mechanical damage to the protein structure and further augmented by secondary damage associated with inflammatory processes occurring several days following the initial insult. In addition, exercise training induces an increase in baseline heat shock protein (HSP) levels, which is dependent on a sustained and currently unknown dose of training and also on the individual’s initial training status [73]. Further studies are necessary to characterize the exercise-induced stress response to exercise protocols. In this respect, marine bioactives could offer a non-pharmacological intervention; the possible correction of pro-inflammatory pathways may prove effective in providing protection against stress oxidative-related diseases and in preserving muscle function during aging.

## 4. Negative Effects of Exercise on Articular Cartilage and the Protective Role of Sea Food: *Stypotriol* and *Hymenialdisine*

The effect of exercise on articular cartilage has been studied in animal models and in humans through various imaging techniques. Joint cartilage maintains the load distribution and joint function under sports activities or prolonged immobilization, thanks to its water content and to the synovial fluid; however, when these factors are reversed, deformed cartilage returns to its former state under normal conditions. Even if moderate exercise contributes to cartilage healing and can decrease the number of cases requiring arthroplasty, on the other side, excessive exercise may be associated with increased cartilage damage or degenerative changes. Despite the presence of osteophytic changes in the joint cartilage of athletes performing mild sports activities, these may not result in osteoarthritis, due to the adaptive feature of joint cartilage, which, unfortunately, is limited in the case of excessive load bearing. Conversely, the risk for osteoarthritis is increased in professional sportsmen exposed to acute repetitive impact and torsional loading [98]. Many studies suggest that participation in sports that subject joints to high levels of impact and torsional loading increases the risk of joint injury and subsequent joint degeneration. This can lead to post-traumatic osteoarthritis, a clinical syndrome caused by trauma-initiated joint degeneration that results in permanent and often progressive joint pain and dysfunction. An evaluation of joint structure and function, muscle strength and neuromuscular function before participating in vigorous physical activity and an immediate diagnosis with appropriate treatment and rehabilitation following joint injuries decrease the risk of subsequent injuries and posttraumatic osteoarthritis [99]. In this sense, nutrition could have a potential preventive and therapeutic role in cartilage health. Some marine bioactives were shown to have a beneficial effect on articular tissue with a positive influence on weight, muscle strength and possibly synovial inflammation. The polycyclic meroditerpenoid, *Stypotriol Triacetate*, isolated from the seaweed, *Stypopodium flabelliforme* [100], showed good anti-inflammatory activity [79]. Stypotriol modulates the cyclooxygenase pathway through inhibition of phospholipase A2, decreasing the secretion of eicosanoids [63]. In addition, it interferes with elastase release, resulting in inhibiting inflammation and reducing elastase-induced cartilage degradation and damage, typical of articular overuse and of diseases, such as osteoarthritis (OA) and rheumatoid arthritis (RA), and responsible for pain and the loss of joint function. In this respect, another interesting marine natural product is the alkaloid, hymenialdisine, isolated from marine sponges, such as *Stylissa massa* [75], and investigated for its inhibitory effect on IL-8, IL-2, IL-1β and TNF-α production [76]. Hymenialdisine blocks some proteins regulating the cellular cycle, such as glycogen synthase kinase-3β, cyclin-dependent kinases and casein kinase 1, through its competition with ATP for binding to these kinases [77]. It also inhibits NF-κB activity, by inhibiting both protein kinase C and I-kB phosphorylation [78], resulting in it being able to interfere with the factors involved in the modulation of gene expression, such as NF-κB. In addition, hymenialdisine was tested on bovine articular cartilage, showing an inhibitory effect on proteoglycan degradation [101]: This promising inhibitory effect on proteoglycan degradation should be investigated in human cartilage and articular damage.

## 5. Sports and Immune System: A Valuable Ally from the Sea

**Figure 2:**
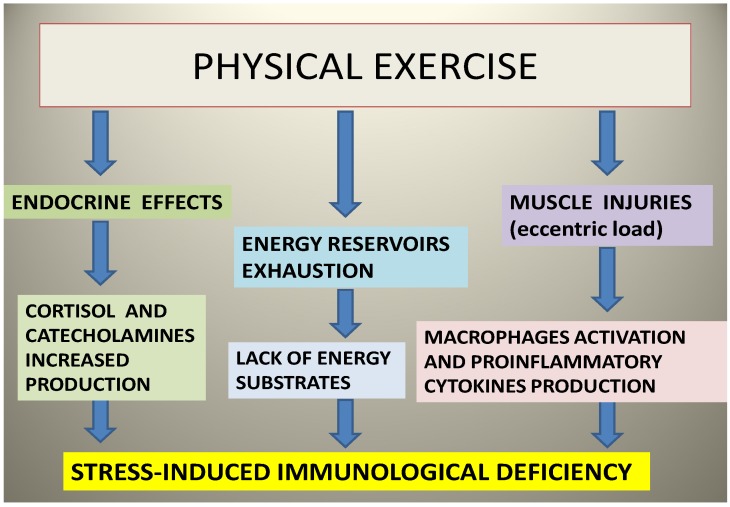
The hormonal effects of physical exercise: the production of cortisol and catecholamines increases, and macrophages are activated and produce proinflammatory cytokines, determining a stress-induced immunodeficiency.

Sports can determine hormonal effects, in particular, the secretion of cortisol and catecholamines. This involves an immune reaction to stress (Figure 2). In addition, exercise depletes the “reservoir” of body energy, mostly represented by glycogen. Another type of immunologic stress is caused by microtraumatic muscle injuries associated with exercise: in particular, a mechanical eccentric stress activates macrophages, with the subsequent release of cytokines. For these reasons, intense exercise causes an immune reaction to stress. This situation can be often recognized through a slight increase in creatine kinase, because of the destruction of the “Z structure” in the muscle [102]. During moderate exercise, significant changes in lymphocyte populations happen: after physical activity lasting 45 min, there is both an increase (six times the physiological blood levels) of natural killer cells, which are important to counter viral infections and neoplasms, and an increase of 20% of the initial value in “CD8-suppressors” [103]. This positive action of moderate exercise on the immune system does not exist after very intense stress, for example in marathon runners, where excessive fatigue is associated with increases in the risk of respiratory tract infections even seven times greater than the control population [104]. Not only in marathon runners, but in general, in the case of excessive training, there is a greater susceptibility to infections, for temporary immunosuppression. In this respect, a valuable aid is represented by some marine natural products with antimicrobial action. For example, epitaondiol exhibited antimicrobial effects against Gram-positive and Gram-negative bacteria, especially against *E. faecalis* [100], and antiviral activity against herpes simplex [94]. The antimicrobial activity of Pacifenol derivatives has previously been reported, too, after testing against some microrganisms, especially against *Pseudomonas aeruginosa* and *Streptococcus enteriditis*. Cyclomarins, three cyclic heptapeptides (A, B and C) belonging to *Streptomyces* sp., isolated from the marine bacterium actinomycete, along the Californian coast, showed antibacterial and anticancer properties [105]. These molecules also showed an ability to kill *Mycobacterium tuberculosis* by targeting its caseinolytic protease, resulting in a promising component of antitubercular drugs [106]. Five other peptides (salinamide A, B, C, D and E) isolated, like cyclomarin, from marine actinomycetes belonging to *Streptomyces sp*. on the surface of the jellyfish, *Cassiopea xamachana*, found in Florida waters [107], had antimicrobial activities. In particular, salinamides A and B, could be used in the treatment of tissue inflammation and some infections, because of their topical anti-inflammatory activity and moderate antibiotic activity against Gram-positive bacteria [108]. This could be exploited to contrast the mild immunodepression typical of overtrained athletes.

## 6. PUFAs as the Ideal Sports Supplement

Polyunsaturated fatty acids (PUFAs) are fatty acids that contain more than one double bond in their backbone. This class includes many important compounds, such as essential fatty acids and those that give drying oils their characteristic property. Polyunsaturated fatty acids can be classified in various groups by their chemical structure: Methylene-interrupted polyenes (omega-3, omega-6, omega-9), which have two or more *cis* double bonds that are separated from each other by a single methylene bridge (CH_2_ unit), conjugated fatty acids and other polyunsaturated fats. The *n*-3 and *n*-6 PUFAs are stored in membrane phospholipids and are responsible for numerous cellular functions, like the cell membrane structure, fluidity, signaling and cell-to-cell interaction [45]. The sea represents the most important source of polyunsaturated fatty acids. In particular, omega-3 marine fatty acids (Table 2) are essential in human nutrition: they also regulate blood pressure, blood clotting, glucose tolerance, inflammatory processes and nervous system function, being helpful in preventing and treating several diseases. In particular, Docosahexaenoic acid (DHA) has been shown to enhance lipid oxidation and insulin sensitivity in skeletal muscle, and it can increase glycolytic capacity in muscle cells. Acid eicosapentaenoic (EPA) and DHA can be found in all fish species, but especially in fatty fish from cold climates, such as salmon, mullet and mackerel. Other important marine sources of omega-3 are algae and crustaceans, such as krill [46]. For example, green seaweeds, like *Ulva pertusa*, are characterized by the presence of hexadecatetraenoic (*n*-3), oleic and palmitic acids. The *n*-3 fatty acid, octadecatetraenoic acid, is abundant in *Laminaria* sp. and *Undaria pinnatifida*, while hexadecatetraenoic acid is prominent in *Ulva* sp. [47]. Omega-3 polyunsaturated fatty acids (PUFAs) have been shown to decrease the production of inflammatory eicosanoids, cytokines and reactive oxygen species, have immunomodulatory effects and attenuate inflammatory diseases. While a number of studies have assessed the efficacy of omega-3PUFA supplementation on red blood cell deformability, muscle damage, inflammation and metabolism during exercise, only a few have evaluated the impact of omega-3 PUFA supplementation on exercise performance. The optimum balance between *n*-3 and *n*-6 fatty acids of macroalgae makes these marine foods that are effective as supplements in a balanced diet for sports [48]. In fact, marine fatty acids have also a stimulating effect on protein synthesis and have been shown to increase fat oxidation, to reduce body weight and to prevent weight gain. The omega-3 modulator effect on the permeability of cell membranes and on the sensitivity to insulin can probably be an ergogenic aid: it could determine the improvement of athletic performance, by making muscle cells more permeable to nutrients, such as glucose and amino-acids. This is supported by the upregulation of the GLUT4 transporter, so that regular consumption of omega-3 could result in being a metabolic stimulator in muscle cells [49]. Many recent studies evidenced a positive outcome of omega-3 consumption in athletes: Omega-3 fatty acid supplementation attenuates oxidative stress and inflammatory markers, such as IL-6, TNF-α, LDH and CK, after eccentric exercise, both in athletes and untrained men [50]; it enhances stroke volume and cardiac output during dynamic exercise, increasing oxygen delivery during exercise, which may have beneficial clinical implications for individuals with reduced exercise tolerance [51]. On the other side, fish oil consumption was shown to reduce heart rate and oxygen consumption during exercise: omega-3 supplementation lowered the heart rate (including peak heart rate) during incremental workloads to exhaustion and lowered the steady-state submaximal exercise heart rate, whole-body O_2_ consumption and blood pressure. Therefore, fish oil may act within the healthy heart and skeletal muscle to reduce both whole-body and myocardial O_2_ demand during exercise, without a decrement in performance [52]. In addition, several studies evidenced the positive effects of omega-3 ingestion on free tryptophan and exercise fatigue [53] and on the attention and reactivity in athletes, improving mood state and reactivity. The reaction time reduction appears to be due to a central nervous system effect, as shown by the reduced latency of movement-related brain macropotentials and Electromyographic EMG activation: this positive effect on the cognitive processes involved in the control of reactivity was demonstrated in karateka engaged in attention tests [54]. Finally, a positive consequence of omega-3 ingestion was evidenced on perceived pain and external symptoms of delayed onset muscle soreness after eccentric exercise in knee extensors, also in untrained men [55].

**Table 2:** Classification of omega-3.

Common Name	Lipid Name	Chemical Name
Hexadecatrienoic acid (HTA)	16:3 (*n*-3)	*all*-*cis*-7,10,13-hexadecatrienoic acid
α-linolenic acid (ALA)	18:3 (*n*-3)	*all*-*cis*-9,12,15-octadecatrienoic acid
Stearidonic acid (SDA)	18:4 (*n*-3)	*all*-*cis*-6,9,12,15,-octadecatetraenoic acid
Eicosatrienoic acid (ETE)	20:3 (*n*-3)	*all*-*cis*-11,14,17-eicosatrienoic acid
Eicosatetraenoic acid (ETA)	20:4 (*n*-3)	*all*-*cis*-8,11,14,17-eicosatetraenoic acid
Eicosapentaenoic acid (EPA, timnodonic acid)	20:5 (*n*-3)	*all*-*cis*-5,8,11,14,17-eicosapentaenoic acid
Heneicosapentaenoic acid (HPA)	21:5 (*n*-3)	*all*-*cis*-6,9,12,15,18-heneicosapentaenoic acid
Docosapentaenoic acid (DPA, clupanodonic acid)	22:5 (*n*-3)	*all*-*cis*-7,10,13,16,19-docosapentaenoic acid
Docosahexaenoic acid (DHA, cervonic acid)	22:6 (*n*-3)	*all*-*cis*-4,7,10,13,16,19-docosahexaenoic acid
Tetracosapentaenoic acid	24:5 (*n*-3)	*all*-*cis*-9,12,15,18,21-tetracosapentaenoic acid
Tetracosahexaenoic acid (nisinic acid)	24:6 (*n*-3)	*all*-*cis*-6,9,12,15,18,21-tetracosahexaenoic acid

It has been suggested that the ingestion of EPA and DHA of approximately 1–2 g/day, at a ratio of EPA to DHA of 2:1, may be beneficial in counteracting muscular fatigue and exercise-induced inflammation and for the overall health of the athlete [109]. However, it is known that high omega-3 PUFA consumption may prolong bleeding time. Thus, attempts should be made to establish an optimal omega-3 fatty-acid dosage to maximize the risk-to-reward ratio of supplementation, since the human data are inconclusive as to whether omega-3 PUFA supplementation at this dosage is also effective in improving performance and not only in attenuating the inflammatory and immunomodulatory response to exercise.

## 7. Other Compounds

### 7.1. Proteins

Proteins are biopolymers of amino acids, some of which are essential for human beings, as they cannot be obtained unless by feeding, because of some deficiency in synthesizing them or in the amount being sufficient. In addition, some proteins, smaller peptides and amino acids have functions that contribute to some health benefits, besides the nutritional benefits. Dietary guidelines state that an adequate daily dietary protein intake for healthy people is 0.9 g protein/kg body weight. According to the US/Canadian Dietary Reference Intakes, this RDA is sufficient to meet the nutrient requirement of nearly all healthy individuals. The US and Canadian Dietetic Association states that protein recommendations for endurance and strength-trained athletes range from 1.2 to 1.7 g/kg/day. This practical recommendation for athletes may reflect a situation where an adaptive advantage of protein intakes higher than recommended protein requirements exists [110]. However, the lack of awareness of some athletes regarding required nutritional practices is reflected by a frequent consumption of protein supplements, amino acid supplements, carnitine, minerals and vitamin supplements and sports drinks to enhance performance in training. An adequate and balanced diet, which meets their higher vitamin and mineral requirements, with sufficient water supply, represents a safe and healthy alternative that can promote peak athletic performance. In particular, during times of high physical activity, an adequate and balanced diet should ensure that the energy and macronutrient needs are met to maintain body weight, to replenish glycogen stores, to provide adequate protein to build and repair tissues and to provide essential fatty acids and fat-soluble vitamins [111]. In crustaceans and mollusks, protein levels can vary from 7% to 23%; therefore, it is worthwhile to look at the protein complement in fish and shellfish. Fish and shellfish muscle proteins are classified into three main groups: sarcoplasmic, myofibrillar and stroma proteins. Sarcoplasmic proteins account for approximately 15%–35% of the total muscle tissue protein. These proteins, which are present in the sarcoplasm, consist mainly of enzymes associated with energy production, such as creatine kinase, aldolase and glyceraldehyde-3-phosphate dehydrogenase. Compositional differences have been reported between fish and mammalian-derived sarcoplasmic protein: for example, fish myoglobin was shown to contain cysteine, while the mammalian equivalent lacks this residue; invertebrate muscle contains paramyosin, a protein not present in vertebrate muscle [112]. Microalgae are sources of valuable amino acids and peptides, too: *C. vulgaris* is rich in proteins with excellent emulsifying properties, which could find valorization as a food complement. Its amino acid profile includes both essential (valine, tryptophan, lysine, methionine, threonine, isoleucine, leucine, phenylalanine) and non-essential amino acids: aspartic acid, serine, glutamic acid, proline, glycine, alanine, cysteine, tyrosine, histidine and arginine [113]. Therefore, microalgae, such as *Arthrospira* and *Chlorella*, because of their richness in protein and their amino acid profile, may be used as nutraceuticals or be included in functional foods to prevent some diseases and damage in cells/tissues, resulting in them being good supplements in the diet of athletes. As a matter of fact, there are already some brand mark pills in the market made of dried *Arthrospira* and *Chlorella*, as it is the case of Hawaiian Pacifica’s Spirulina [15]. Until now, most of the biological effects of marine-derived protein hydrolysates and peptides have been seen *in vitro* or in animal models. A limited number of human studies has been performed to date, so that human intervention trials need to be performed to demonstrate the efficacy of their bioactivities and more a detailed understanding of the mechanisms by which different peptides and amino acid may mediate their physiological effects.

### 7.2. Enzymes and Vitamins

Amino acids are also the constituents of some other biocompounds, such as hormones and enzymes, that along with proteins, are used for growth, the replacement of damaged tissues, which is really important in the time recovery of athletes after sports injuries, and other functions responsible for maintaining human health. Enzymes, such as superoxide dismutase, are metalloenzymes that can contain iron, manganese or zinc and function against free radicals as antioxidants, defending cells against oxidative damage. Different kinds of these enzymes are also produced by microalgae, such as *Anabaena* and *Porphyridium*. Carbonic anhydrase is another metalloenzyme present in the blood cells, which catalyzes the reaction that converts CO_2_ into carbonic acid and bicarbonate ions in the tissues. It was found in the marine microalgae, *Isochrysis galbana* [15]. In addition, *Haslea ostrearia* is particularly rich in vitamin E, while *P. cruentum* is another microalga rich in vitamins C and E and provitamin A (β-carotene). *D. salina* not only produces β-carotene (provitamin A), but also thiamine, pyridoxine, riboflavin, nicotinic acid, biotin and tocopherol [44].

### 7.3. GABA

Porphyridium is one of the marine microalgae that produces gamma-amino butyric acid (GABA), an amino acid that is the main inhibitor neurotransmitter for the central nervous system of adult mammals [15]. It is responsible for regulating neuronal excitability and also for muscle tone. A great deal of effort has been expended in attempting to define the role of GABA in mediating the transmission and perception of pain. The pursuit of this question has been stimulated by the fact that GABAergic neurons are widely distributed throughout the central nervous system, including regions of the spinal cord dorsal horn known to be important for transmitting pain impulses to the brain. In addition, GABA neurons and receptors are found in supraspinal sites known to coordinate the perception and response to painful stimuli, and this neurotransmitter system has been shown to regulate the control of sensory information processing in the spinal cord. The discovery that the GABA receptor agonists show antinociceptive properties in a variety of animal models of pain has provided an impetus for developing such agents for this purpose. It has been shown that GABA receptor agonists, as well as inhibitors of GABA uptake or metabolism are clinically effective in treating this symptom, and the stimulation of the GABA receptor could be of benefit in the management of pain [74].

## 8. Conclusions

Increased muscle oxidative stress and inflammatory responses among athletes have been reported consistently. In addition, it is well known that exhaustive or exercise that on is unaccustomed to can lead to muscle fatigue, delayed-onset muscle soreness and a decrement in performance. Intense physical exercise determines increased oxidative stress and a tendency toward inflammation, because of a greater respiratory activation chain and the insufficient disposal of excess oxygen. Consequently, increasing antioxidant intake would reduce oxidative stress, with a subsequent benefit to athletes. According to the American Dietetic Association and to the American College of Sports Medicine, physical activity, athletic performance and recovery from exercise are improved by optimal nutrition [114]. Physiological adaptations produced as a consequence of physical exercise lead to the necessity to increase the caloric (considering the greater energy output), protein (because of the increased trophic needs of the organism during and after exercise) and antioxidant intake (on the basis of the higher oxidative stress). Athletes have to follow a diet that is adequate for greater energy output and for higher metabolic turnover. Supplementation varies according to different sports practices and individual athletes; nevertheless, antioxidant, anti-inflammatory and cartilage-protecting bioactives could be useful in every kind of sport, because of both health reasons and sports performance. The sea is a rich source of useful compounds with new chemical structures and pharmacological effects: significant immunomodulation (against allergy), anti-inflammatory (and as a consequence, analgesic), antibacterial and antiviral activities [115]. It may represent a convenient and practical means of providing the special nutrient requirements for exercise, and it may be used to prevent nutritional deficiencies that commonly occur among athletes. The role of food in improving health has been recognized, activating the development of new classes of food, known as functional foods [116], which could improve the quality of life and performance and decrease the risk of illness. Fish oils and marine bacteria are known to be excellent sources of omega-3 fatty acids (whose importance in the treatment of arthritis has been greatly investigated [117], assessing their analgesic effects in joint pain), while seaweeds and crustaceans seem to contain powerful antioxidants, such as carotenoids and phenolic compounds [118]. Although several points of discussion still exist, such as the necessary doses and efficacy, not only in the antioxidant effect, but also in improving physical performance, the question of whether antioxidants have a protective role in exercise-induced muscle damage can be answered affirmatively. Numerous human studies indicate that antioxidant supplementation can be recommended to individuals performing regular physical exercise. Moreover, trained individuals have an advantage compared with untrained individuals, as training results in the increased activity of several major antioxidant enzymes and the overall antioxidant status [119]. The pharmacology of structurally-characterized products extracted from marine seafood (fishes, crustaceans, sponges, fungi, algae and bacteria) has recently been discussed [120]. Antibacterial, antiviral, antifungal, antiprotozoal and anti-tuberculosis pharmacological activities were reported for about 100 marine natural compounds. In addition, about 150 marine metabolites were showed to possess anti-inflammatory effects, to enhance the immune and nervous system and to interact with a variety of receptors and molecular targets, as well. In particular, marine pharmacology studies are now focusing on the *in vitro* anti-inflammatory effect through the inhibition of NF-κB by ethanolic extracts from the brown alga, *Ishige okamurae* [121]; on the antioxidant activity in phenolic compounds isolated from the marine alga, *Halimeda macroloba*, that protected against chemically-induced injury *in vivo* (animal studies on rat) [122]; on the high antioxidant property in methanolic extracts of the Korean red alga, *Polysiphonia morrowii*, that showed protection against radical-induced DNA damage *in vitro* [120]; on the antioxidant effects of polysaccharides from a marine fungus, *Penicillium* sp., against superoxide and hydroxyl radicals; on the antioxidant activities of the phosphorylated, acetylated and benzoylated metabolites of the marine red alga, *Porphyra haitanensis*, shown *in vitro* [120]; on the human neutrophil anti-elastase activity of sulfated polysaccharides from the red alga, *Delesseria sanguinea* [123]; and finally, on the acceleration of skin wound healing by amino acids extracted from the mollusk, *Rapana venosa* [124], suggesting a possible therapeutic use in sports injuries. In this respect, we could theorize about the protective effect of marine bioactives, especially in athletes. In conclusion, marine bioactives could potentially develop as functional foods influencing the pathogenesis and the clinical course of several inflammatory diseases [125], and in the future, their introduction into the human habitual diet could lead not only to a reduction in the incidence and severity of many disorders [126,127], but also to a practical and valuable aid for athletes’ health and, consequently, performance. At present, the great potential of marine compounds, as such or as extracts, for applications in different areas, such as human nutrition, as anti-inflammatory, antiallergic and analgesic agents, is gaining more attention in the literature (Table 1). Actually, some researchers also highlighted the fact that some marine unicellular algae, such as *Porphyridium* and *Rhodella*, and cyanobacteria, such as *Arthrospira*, can produce sulfated polysaccharides, which have already found application as antiviral agents, either *in vivo* or *in vitro* [128], but also as nutraceuticals [129], as agents to prevent tumor cell growth [130], as therapeuticals [131] or even as ion exchangers [15]. However, there are many other compounds produced by unicellular marine algae that have already found or could have some health applications; for example, sterols, pigments, proteins and enzymes, vitamins and several other substances. Ongoing studies are showing that marine microalgae and cyanobacteria, which synthesize several high-value compounds, can grow under controlled conditions. Therefore, these valuable biochemical compounds have already proven their wide range of applications, but there are further potentialities yet to be explored. It would be very challenging to use these substances in order to enhance athletes’ performance with no contraindications and side effects. Furthermore, future human studies investigating the efficacy of marine bioactives supplementation in exercise-trained individuals should consider an exercise protocol of sufficient duration and intensity to produce a more robust oxidative and inflammatory response.
